# Genetic Architecture of MAPT Gene Region in Parkinson Disease Subtypes

**DOI:** 10.3389/fncel.2016.00096

**Published:** 2016-04-11

**Authors:** Esterina Pascale, Maria Elena Di Battista, Alfonso Rubino, Carlo Purcaro, Marcella Valente, Francesco Fattapposta, Giampiero Ferraguti, Giuseppe Meco

**Affiliations:** ^1^Department of Medical-Surgical Sciences and Biotechnologies, Sapienza UniversityRome, Italy; ^2^Department of Neurology and Psychiatry (Parkinson’s Centre), Sapienza UniversityRome, Italy; ^3^Research Centre of Social Diseases (CIMS), Sapienza UniversityRome, Italy; ^4^Department of Cellular Biotechnologies and Hematology, Sapienza UniversityRome, Italy

**Keywords:** microtubule-associated protein tau gene, h1 subhaplotype, Parkinson disease, non tremor dominant Parkinson disease, tremor dominant Parkinson disease, Parkinson subtype

## Abstract

The microtubule-associated protein tau (MAPT) region has been conceptualized as a model of the interaction between genetics and functional disease outcomes in neurodegenerative disorders, such as Parkinson disease (PD). Indeed, haplotype-specific differences in expression and alternative splicing of MAPT transcripts affect cellular functions at different levels, increasing susceptibility to a range of neurodegenerative processes. In order to evaluate a possible link between MAPT variants, PD risk and PD motor phenotype, we analyzed the genetic architecture of MAPT in a cohort of PD patients. We observed a statistically significant association between the H1 haplotype and PD risk (79.5 vs 69.5%; χ^2^ = 9.9; OR, 1.7; 95% CI, 1.2–2.4; *p* = 0.002). The effect was more evident in non tremor dominant (TD) PD subjects (NTD-PD) (82 vs 69.5%; χ^2^ = 13.6; OR, 2.03; 95% CI, 1.4–3; *p* = 0.0003), while no difference emerged between PD subgroup of tremor dominant patients (TD-PD) and control subjects. Examination of specific intra-H1 variations showed that the H1h subhaplotype was overrepresented in NTD-PD patients compared with controls (*p* = 0.007; OR, 2.9; 95% CI, 1.3–6.3). Although we cannot exclude that MAPT variation may be associated with ethnicity, our results may support the hypothesis that MAPT H1 clade and a specific H1 subhaplotype influence the risk of PD and modulate the clinical expression of the disease, including motor phenotype.

## Introduction

The microtubule-associated protein tau (MAPT) is a phosphorylated protein primarily expressed in the brain, where it assists in stabilization of the cytoskeleton and axonal transport in neurons. The human gene encoding MAPT lies on chromosome 17q21, within a ~900 kb ancestral genomic inversion that generates a ~1.8 megabase (Mb) region of linkage disequilibrium (LD) defined by two extended haplotypes, referred to as H1 and H2 (Baker et al., 1999). In contrast to H2, the H1 haplotype is evolutionarily dynamic and contains a number of variation composed of single nucleotide polymorphisms (SNP) highly correlated with each other (Pittman et al., 2005).

Dominantly inherited mutations in MAPT were formerly associated with forms of frontotemporal dementia and parkinsonism linked to chromosome 17, first providing evidence of a link between tau dysfunction and neurodegeneration (Hutton et al., 1998; Spillantini et al., 1998). Beside dominantly inherited diseases, the most common H1 haplotype has been linked not only to tauopathies such as Alzheimer’s disease (AD), progressive supranuclear palsy (PSP) and corticobasal degeneration (CBD; Myers et al., 2005; Pittman et al., 2006) but also to the most common synucleinopathy such as Parkinson disease (PD), providing mostly positive albeit partially conflicting results (see Zabetian et al., 2007 for summary). However, the impact of H1 haplotype as a risk factor for PD has been confirmed in GWAS studies (Simón-Sánchez et al., 2009; Edwards et al., 2010) and meta-analysis by the PDGene forum^1^ (Lill et al., 2012).

Given the convergent data indicating a role of MAPT locus in PD, recent lines of research have moved to refine the potential effect of H1 haplotype into phenotypic traits of the malady (Huang et al., 2015; Davis et al., 2016; Wang et al., 2016), such as the progression of cognitive deficits (Williams-Gray et al., 2009; Seto-Salvia et al., 2011) and motor clinical subtype (Di Battista et al., 2014).

Moreover, taking into account that the true allele risk associated with neurodegenerative disorders could reside at any position within an approximately 900 kb region, that includes genes other than MAPT, the H1 haplotype has been partitioned into several H1-specific subhaplotypes in order to more precisely map the disease-associated region. Currently fine-mapping studies of the MAPT H1/H2 clades have identified specific subhaplotypes associated with AD (Myers et al., 2005) and PSP (Pittman et al., 2005), while mostly conflicting results have been described with PD (Fung et al., 2006; Tobin et al., 2008; Vandrovcova et al., 2009; Seto-Salvia et al., 2011).

Therefore, we sought to analyze the genetic architecture of MAPT in a cohort of PD patients where we had formerly observed an association between H1 homozygosity and non-tremor dominant (NTD) PD subtype, and whether specific variants of the H1 clade were linked with clinical phenotypes.

## Materials and Methods

### Subjects

A total of 197 unrelated control subjects (mean age: 68.2 ± 18.8, 67% male) and 181 unrelated sporadic PD patients (mean age: 70.9 ± 8.4 years, 56% male, mean age of diagnosis: 60.7 years) were consecutively recruited from March 2011 to July 2012 for the present study. This study cohort has been formerly described in a published study (Di Battista et al., 2014) that analyzed PD motor subtypes in H1 homozygote vs. H2 carriers, no control subjects were enrolled for our previous report. All patients were of European ancestry and originated from the regions of Central and Southern Italy. Patients were selected from the Parkinson outpatient center of the Sapienza University of Rome and fulfilled the UK Brain Bank criteria for PD. Patients with signs of atypical parkinsonism, doubtful response to dopaminergic replacement therapy, dementia (Mini-Mental State Examination score < 24) or unreliable clinical data (disease duration ≤ 3 years, less than three clinical assessments) were not included.

### Clinical Assessment

For the assignment of clinical subtype, the patients underwent at least three clinical assessments by two expert neurologists in movement disorders during the study period, moreover, clinical notes were reviewed to obtain retrospective data on clinical onset. The UPDRS III performed in the last visit is reported. The patients were classified into two clinical subtypes, by applying the criteria published in a previous study (Selikhova et al., 2009): (a) tremor dominant (TD), i.e., patients with tremor as the only motor sign at onset or tremor as the prominent motor symptom according to the UPDRS part III; (b) NTD, i.e., patients with predominant rigidity and bradykinesia but no tremor or only mild tremor at rest. Only patients with at least 3 years of disease duration were enrolled for the study in order to obtain a reasonable depiction of the clinical subtype.

Clinicians were blinded of patients MAPT background during the study examinations. The patients’ clinical and demographic data are shown in Table 1. The controls were selected among blood donors. Written informed consent to the study was obtained from all the PD patients and control subjects. The study was approved by the local ethics committee of Sapienza University of Rome.

**Table 1:** **Demographic and clinical characteristics of patients and controls**.

Characteristic	Controls *N* = 197	PD *N* = 181	NTD-PD *N* = 135	TD-PD *N* = 46
Male sex, No. (%)	67% (132)	56% (102)	58% (78)	52% (24)
Age (Mean ± SD)	68.2 ± 18.8	71.8 ± 8.6	71.7 ± 8.5	71.9 ± 9.2
Age at onset (Mean ± SD)	NA	60.5 ± 8.9	60.4 ± 9.2	61.0 ± 7.8
Disease duration (Mean ± SD)	NA	10.4 ± 4.8	10.4 ± 5.1	10.3 ± 4.1
UPDRS III score	NA	19.5 ± 9.6	20.6 ± 9.9	16.5 ± 8.1

### Genetic Analyses

DNA from peripheral blood was isolated using standard procedures. MAPT haplotype was determined by testing for the presence of a 238 bp deletion between exons 9 and 10 (*del-In9)*, which is characteristic of the H2 haplotype. The *del-In9* polymorphism was amplified by polymerase chain reaction (PCR) and separated on 6% native polyacrylamide gel and then visualized after ethidium bromide staining by UV transillumination. The amplification reaction was set to a volume of 25 μl containing 1.5 mM MgCl_2_, 200 μM dNTP, 50 mM KCl, 10 mM Tris-HCl, pH 8.3, 0.25 μM of each primer and 1 U of Promega Taq DNA polymerase, 30 amplification cycles were performed. PCR conditions for *del-In9* were: 94°C for 30 s, 63°C for 30 s and 72°C for 30 s The forward primer was 5′-GTTTCCACTGTTTCCAGAGTTCC and the reverse primer was 5′-TTTTACAATCTCAGCCCCTAGC. H1 yields a 574 bp product and H2 a 336 bp product under these conditions.

The SNPs rs1467967, rs242557, rs3785883, rs2471738, rs7521 were detected by means of the restriction fragment length polymorphism PCR (PCR-RFLP) method. To amplify by PCR the MAPT haplotype SNPs of interest, oligonucleotide primer pairs were designed using Primer3 software^2^. A volume of 10 μl of PCR product for each SNPs was digested by 1 U of the corresponding restriction endonuclease: rs1467967 (*DraI*); rs242557 (*ApaHI*); rs3785883 (*BsaHI*); rs2471738 (*BstEII*); rs7521 (*PstI*). All nucleases were from New England Biolabs and the reaction volume of 20 μl was incubated for 2 h at the temperature indicated by the manufacturer. The sequences of primers for PCR reactions, restriction enzymes used for RFLPs and size of fragments are shown in Table 2. For the SNPs rs1467967, rs242557 and rs2471738 a multiplex PCR reaction was performed in a volume of 50 μl, at the following settings: 94°C for 30 s, 60°C for 30 s and 72°C for 30 s. PCR conditions for the rs3785883 were: 94°C for 30 s, 54°C for 30 s and 72°C for 30 s. The SNP rs7521 was amplified using the following parameters: 94°C for 30 s, 62°C for 30 s and 72°C for 30 s. Genotyping accuracy was confirmed randomly by DNA sequencing.

**Table 2:** **Primer sequences and restriction enzymes used for detection of the investigated polymorphisms**.

Gene polymorphism (alleles)	PCR primers (5′−3′)	Enzyme (fragment bp)
rs1467967 (G/A)	Fwr: CACAGCCACCCTCCCTCTAAC	*DraI*
	Rev: GGCTCCACCCTTCAGTTTTGGA	(267/ 186, 81)
rs242557 (A/G)	Fwr: CTTGATGATGCATGGACCTCTC	*ApaHI*
	Rev: TTGACAGTACCCACGACACGTG	(211/ 139, 72)
rs3785883 (A/G)	Fwr: CCATCACCTTGTCAGAAACTC	*BsaHI*
	Rev: AGCCATGTGGTAGCCTCAG	(277/ 164, 113)
rs2471738 (T/C)	Fwr: CTCTCTGGACCCTCATCCACC	*BstEII*
	Rev: GAGAACCGAATGAGGACTGGAA	(170/ 104, 66)
rs7521 (G/A)	Fwr: ACCTCTGTGCCACCTCTCAC	*PstI*
	Rev: AGGTGAGGCTCTAGGCCAGT	(231/ 160, 71)

*Fwr, forward primer; Rev, reverse primer*.

### Statistical Analysis

We assessed each SNP for Hardy-Weinberg equilibrium in cases and control subjects. For all markers, Fisher’s exact test was used to test for allele frequencies association between cases (PD, NTD-PD, and TD-PD) and controls. *P*-values were considered significant at *P* < 0.05.

The program FAMHAP Ver.19^3^ (Herold and Becker, 2009) was used to reconstruct H1 subhaplotypes and calculate haplotypes frequencies. For comparison reasons, subhaplotypes with frequencies <5% were included in the analysis only if observed at a higher frequency (>5%) in one of the other groups. We first performed a global likelihood ratio test to assess whether the overall subhaplotype frequency distribution differed between cases and control subjects. In instances in which the overall distribution significantly differed we examined the effect of each individual subhaplotype compared with all others. We calculated pairwise LD (measured as D′) between subhaplotypes in cases and control subjects, and created a graphic representations of the data using Haploview 4.1^4^ (Barrett et al., 2005). A Bonferroni correction was used to take into account multiple testing.

## Results

Following the classification procedure, we found 46 TD-PD and 135 NTD-PD patients. Patient groups (NTD-PD vs. TD-PD) were comparable with regard to age, age at onset and disease duration. A statistically significant difference in motor performance assessed with UPDRS part III at last visit was observed between the two PD groups (NTD-PD 20.6 ± 9.9 vs. TD-PD 16.5 ± 8.1; *p* < 0.05; Table 1).

Six MAPT htSNPs were tested for association in all patients and control group. Allele frequencies for all SNPs were in Hardy-Weinberg equilibrium, except for rs2471738, for which a marginally significant deviation was seen in cases (*p* = 0.02) but not in control subjects (*p* = 0.9). To test the relation between H1-SNPs and PD risk, we first performed a single-locus analysis of the MAPT genetic variants. Results are shown in Table 3. A significant overrepresentation of the H1 allele in the entire PD group (comprising both NTD-PD and TD-PD) compared with controls (79.5 vs. 69.5%; χ^2^ = 9.9; OR, 1.7; 95% CI, 1.2–2.4; *p* = 0.002) was detected. The association was greater in the PD subgroup of NTD patients compared with controls (82 vs. 69.5%; χ^2^ = 13.6; OR, 2.03; 95% CI, 1.4–3; *p* = 0.0003) and remained significant after correction for multiple testing (*p*_correct_ = 0.008), while no statistically significant difference was disclosed between PD subgroup of TD patients and control subjects (72 vs. 69.5%; χ^2^ = 0.17; OR, 1.1; 95% CI, 0.7–1.9; *p* = 0.7). Among the other SNPs, only the rs3785883 was marginally statistical significant in the NTD-PD subgroup compared with control subjects (χ^2^ = 4.3; OR, 1.5; 95% CI, 1.02–2.3; *p* = 0.044), but this difference did not remain significant after statistical correction considering the number of SNPs analyzed.

**Table 3:** **Microtubule-associated protein tau (MAPT) single single nucleotide polymorphisms (SNPs) association results**.

			Major allele frequency			
Variant	Location in MAPT	Major allele	CO	PD (*p*-value) OR (95%CI)	NTD-PD (*p*-value) OR (95%CI)	TD-PD (*p*-value) OR (95%CI)
rs1467967	5′Exon 1	A	59	60 (0.88)	63 (0.37)	51 (0.16)
				1.02 (0.7–1.3)	1.16 (0.8–1.6)	0.7 (0.41–1.1)
rs242557	5′Exon 1	G	68	66 (0.53)	63 (0.2)	74 (0.31)
				0.9 (0.67–1.2)	0.8 (0.58–1.1)	1.3 (0.8–1.2)
rs3785883	Intron 3	G	83.5	78 (0.064)	77 (0.044)	81.5 (0.64)
				0.7 (0.5–1.02)	0.6 (0.45–0.98)	0.87 (0.5–1.6)
rs2471738	Intron 9	C	80	77 (0.4)	76 (0.25)	81.5 (0.77)
				0.87 (0.6–1.2)	0.8 (0.5–1.2)	1.12 (0.6–2)
del-in9	Intron 9	H1	69.5	**79.5 (0.002)**	**82 (0.0003)**	72 (0.7)
				1.7 (1.2–2.4)	2.03 (1.4–3)	1.1 (0.7–1.8)
rs7521	3′Exon 14	G	55	53 (0.6)	52 (0.47)	55 (1)
				0.92 (0.7–1.2)	0.89 (0.6–1.2)	1.03 (0.6–1.6)

*Abbreviations: CO, controls; PD, Parkinson Disease; PD-NTD, Parkinson Disease Non Tremor Dominant; PD-TD, Parkinson Disease Tremor Dominant; OR, odds ratio; 95% CI, Confidence Interval. Boldface values indicate significant results after Bonferroni correction for multiple testing (p_correct_ = 0.008)*.

To clarify the association found between PD and the MAPT H1 variation and to assess whether any of the H1 subclades previously described (Myers et al., 2005; Pittman et al., 2005) could be influencing PD risk or motor phenotypes, we performed a haplotype association study comparing MAPT subhaplotype frequencies between the different group of patients and controls. On the H1 background, 20 subhaplotypes were identified with a frequency of ≥1% and only those with frequencies >5% in at least one of the groups in study were considered for the analysis. A total of seven subhaplotypes were selected (Table 4). Significant overall differences in selected subhaplotype frequencies (defined by the tagging SNPs rs1467967, rs242557, rs3785883, rs2471738, *del-in9*, rs7521) were found between PD patients and controls (*p* = 0.014), between NTD-PD and controls (*p* = 0.0005) and between TD-PD vs. NTD-PD (*p* = 0.035), while no statistically significant difference was detected between controls and TD-PD patients (*p* = 0.35).

**Table 4:** **MAPT haplotype association results**.

		Haplotype frequency			
Haplotype ID	Haplotype variants^a^	CO	PD	NTD-PD	TD-PD
H2a	AGGCdelG	21	16.7	13^b^	26^c^
H1b	GGGCinsA	17	17	16	24
H1c	AAGTinsG	7	8.8	10.5	5
H1d	AAGCinsA	3	5.3	5.6	5.3
H1e	AGGCinsA	6.3	8.2	9.3	3.7
H1h	AGACinsA	2.6	6.7^d^	**7.4^e^**	5.3
H1i	GAGCinsA	8	5	4.6	5.3
			0.015	0.0005	0.35 (0.035)

*Abbreviations: CO, controls; PD, Parkinson Disease; PD-NTD, Parkinson Disease Non Tremor Dominant; PD-TD, Parkinson Disease Tremor Dominant; MAPT, microtubule-associated protein tau; OR, odds ratio; 95% CI, Confidence Interval. ^a^Alleles for the SNPs defining the haplotype are given in the 5′ to 3′ order as follows: rs1467967, rs242557, rs3785883, rs2471738, del-In9, and rs7521. ^b^0.024, OR 0.6; 95%CI 0.4–0.9: NTD-PD vs. CO. ^c^0.018, OR 0.5; 95%CI 0.28–0.87: NTD-PD vs. TD-PD. ^d^0.013, OR 2.6; 95%CI 1.2–5.5: PD vs. CO. ^e^0.007, OR 2.9; 95%CI 1.3-6.3; NTD-PD vs.CO. (Boldface value indicate significant result after Bonferroni correction for multiple testing, p*_correct_ = 0.00714). *Numbers on the last line indicate p values that result from global haplotype frequency comparison between each group of patients and controls, while the p value in brackets results from comparison between TD-PD and NTD-PD patients*.

The MAPT H2a haplotype was significantly underrepresented in the NTD-PD subgroup compared with controls (*p* = 0.024; OR, 0.6) and with TD-PD patients (*p* = 0.018; OR, 0.5).

Detailed analyses showed a significant difference in the frequency of the H1h subhaplotype in the PD group (*p* = 0.013; OR, 2.6) compared with controls. However, the difference was greater in the subgroup of NTD-PD patients (*p* = 0.007; OR, 2.9). After correction for the number of haplotypes analyzed only this last difference remained statistically significant.

**Figure 1:**
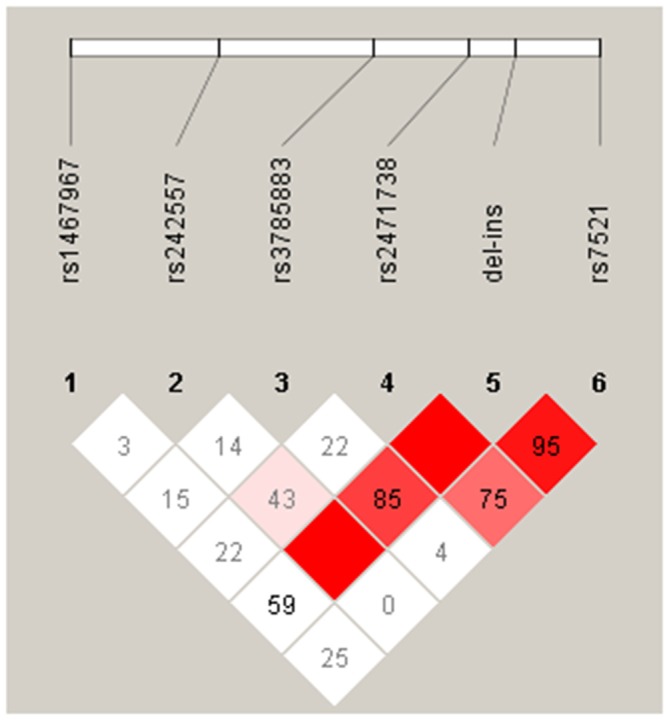
**Linkage disequilibrium (LD) between the microtubule-associated protein tau (MAPT) H1 genotyped single nucleotide polymorphisms (SNPs) in our PD group.** The relative position of the MAPT H1 tagging SNPs, is shown (top). Within each diamond the pairwise standardized coefficient of LD (D′ values × 100) are presented.

The pairwise linkage disequilibrium analysis (LD) for all SNPs was performed in the control and PD groups. The LD plot showed in the Figure 1, indicates in PD patients, that alleles at rs242557, rs3785883, rs2471738 and rs7521 are in strong LD with the *del-in9* (marker 5), but are not in strong linkage with each others, indicating that these markers are H1 specific.

## Discussion

Analyzing the role of MAPT locus in neurodegenerative disorders, such as PD, represents an effort to elucidate the interaction between genetics and functional disease outcomes. Although several studies including ours have found an association between the H1 haplotype and PD, the functional role of this variant still remains to be identified.

This article aimed at refining the MAPT role in PD by examining the architecture of the entire gene in order to determine its possible associations with PD, and PD motor phenotypes, in a cohort of patients of Italian ancestry. Our results are consistent with the growing body of evidence that supports the MAPT H1 haplotype as a risk factor for sporadic PD. Moreover, we observed a peculiar risk distribution of MAPT haplotype, and H1 subhaplotype, according to PD motor phenotype. Indeed, while NTD-PD subgroup showed a statistically significant overrepresentation of H1 clade, no differences were observed between TD-PD subgroup and control subjects. Among the other SNPs analyzed, the rs3785883 polymorphism was nominally significant, exclusively in the NTD-PD subgroup compared with controls. This finding is consistent with a previous study where a moderate association at SNP rs3785883 was also found in a Greek cohort of PD patients (Fung et al., 2006). With regard to MAPT subhaplotype, we found that H1h was associated with PD and this association remained statistically significant after correction for multiple testing when NTD-PD subgroup was considered. To our knowledge this is the first study comprehensively assessing MAPT locus in PD according to clinical motor subtype and the strong point of this study is the regular motor clinical assessment for all patients included in the cohort.

The results of our study potentially raise two orders of consideration: the first report of a significant PD risk of the H1 haplotype in a PD subgroup, namely NTD-PD and the original finding of an association between the H1h subhaplotype and the same PD clinical subtype, in a cohort of Caucasian European ancestry.

At present, phenotype-genotype association studies that analyze the role of MAPT haplotypes on PD are mostly focused on cognitive profiling. These researches overall indicate an involvement of H1 haplotype on specific cognitive domains such as memory and visuo-spatial functions (Williams-Gray et al., 2009). According to these studies, MAPT variation influences cognition and the function of specific brain circuitry even in early phases of PD (Nombela et al., 2014) and even in healthy control subjects (Winder-Rhodes et al., 2015). Although the relationship between MAPT haplotype and cognitive functions remains to be determined since no specific regional degeneration or neurochemical alterations have been provided, the effect seems to be detectable even in relative small number of subjects.

Unlike many other quantitative phenotypic traits, there are now evidences suggesting that clinical motor phenotype of PD may not represent a mere semiological matter. Indeed, while the TD-PD patients could be considered a subgroup with a benign clinical course and a slower process of degeneration at least for the most part of the disease course (Selikhova et al., 2009, 2013; Eggers et al., 2012; Deuschl, 2013), NTD-PD are likely more prone to develop a series of motor and non motor complaints, inherent to the spreading of the degenerative process (Rosenberg-Katz et al., 2013; Zhang et al., 2013; Herman et al., 2014; Solla et al., 2015).

Interestingly, the contribution of MAPT gene in motor impairment has been described in a large community-based cohort of neurologically healthy aging individuals (Shulman et al., 2014), where an association between H1 haplotype and mild parkinsonian signs, especially bradykinesia, has been observed without evidence of PD hallmark at pathological assessment. The authors speculated that neuroanatomical dysfunction of cortico-nigro-striatal pathways, different from those classically observed in PD, may contribute to the development of parkinsonian signs. Therefore, given the prevailing view of H1 haplotype as a genetic risk factor for neurodegeneration, we can speculate that H1 background may partake to the expression of a PD clinical motor subtype associated with increased functional disability.

On the other hand, given the overall underrepresentation of H2 haplotype in a range of neurodegenerative disorders and assuming that this haplotype is associated with more efficient brain function, our observation of a significant overrepresentation of H2 in TD-PD may indicate a protective role of the H2 haplotype. Some studies assessed the role of MAPT variants in gene expression (Hutton et al., 1998; Spillantini et al., 1998; Caffrey et al., 2006; Pittman et al., 2006). Some authors reported that the disease risk conferred by MAPT variants could be related to a higher total or 4R tau levels. Additionally, a protective effect of MAPT H2-haplotype due to an increase espression in N-terminal exon-containing MAPT transcripts has been speculated. Indeed, recent work suggested that N-terminal transcripts may play a role in the regulation of tau solubility, inhibiting tau polymerization (Horowitz et al., 2006).

Nevertheless, although several lines of evidence, including basic researches, pathological findings and genotype-phenotype association support the role of MAPT haplotypes in PD, the mechanistic model of this link remains to be determined. Moreover, the pathological findings related to MAPT background in neurodegenerative disorders are partially conflicting. Indeed, while some studies conducted on PSP human brain indicate that H1 haplotype does not affect the pathological or biochemical phenotypes (Liu et al., 2001), others found an higher expression of 4R-tau from the H1 haplotype compared to H2. Other authors observed that the MAPT H1 haplotype enhances the overall α-synuclein deposition type pathology in dementia with Lewy Body (Colom-Cadena et al., 2013) and Alzheimer’s disease (Wider et al., 2012).

However, no comprehensive assessments of α-synuclein burden or Alzheimer-like pathology according to MAPT haplotype are currently available in PD; therefore, systematic and well-powered analysis exploring the functional outcomes of MAPT region variations remain mandatory. Furthermore, we found that a specific subhaplotype, the H1h variant, was overrepresented in our PD population, more significantly in NTD-PD patients.

A number of studies have been performed for association of MAPT subhaplotypes variability and PD with inconsistent results (Fung et al., 2006; Vandrovcova et al., 2009; Seto-Salvia et al., 2011). Moreover, other studies analyzed just few of the SNPs described to characterize the H1 specific subhaplotypes (Fidani et al., 2006; Winkler et al., 2007; Das et al., 2009; Refenes et al., 2009; Huang et al., 2015; Wang et al., 2016).

Given the concern that H1h variants may be driven by the genetic architecture related to ethnicity (Fung et al., 2006), it is worth considering that a biological link between such variant and neurodegenerative process has been formerly reported. Indeed, it has been found in AD that the A-allele of the rs3785883 SNP is associated with increased cerebrospinal fluid (CSF) tau levels and tau mRNA expression (Kauwe et al., 2008). Moreover, the authors observed that the MAPT genetic variation defined as H1h subhaplotype showed significant elevation of CSF tau compared with the H2 haplotype. The contribution of the A-allele of the rs3785883 to tau expressions in AD has been subsequently confirmed in a larger series (Allen et al., 2014). Therefore, our findings may be congruent with, and complementary to these reports considering that NTD-PD patients showed significantly higher levels of CSF tau protein and tau/beta index if compared to TD-PD (Jellinger, 2012; Přikrylová Vranová et al., 2012).

If confirmed, this result may indicate that a specific H1 subhaplotype increases the risk of developing a NTD-PD disease, at least in populations of South European ancestry. Our study supports the hypothesis that genetic variability in the MAPT region is involved in PD susceptibility and may contribute to PD phenotypic expression, confirming that large-scale evaluation in different populations could be relevant to understand the role of population-specific heterogeneity.

The main limitation of this study is the relative small sample size; due to this major issue the results should be interpreted with caution until further studies in larger series of patient will ascertain the significance of specific subhaplotypes in PD clinical traits.

Given the findings that H1 may act synergistically with other gene variants in determining risk for PD, future researches should concern with gene–gene interactions to provide critical insights into mechanisms of disease susceptibility.

Nevertheless, the genetic architecture of MAPT in determining PD phenotypic expression as well as the possible functional effect of H1h subhaplotype deserves attention and replication in larger series.

## Author Contributions

Study conception and design: EP, MEDB, AR, GM. Acquisition of the data: EP, MEDB, AR, CP, MV, FF. Analysis and interpretation of the data: EP, MEDB, GF, AR. Drafting of manuscript: EP, MEDB, AR, GF. All authors read and approved the final manuscript.

## Conflict of Interest Statement

The authors declare that the research was conducted in the absence of any commercial or financial relationships that could be construed as a potential conflict of interest.

## Footnotes

^1^http://www.pdgene.org/

^2^http://sourceforge.net/projects/primer3/

^3^http://famhap.meb.uni-bonn.de/

^4^https://www.broadinstitute.org/scientific-community/science/programs/medical-and-population-genetics/haploview/downloads
